# DIETARY NITRATE SUPPLEMENTS AND AEROBIC EXERCISE MODIFY FRAILTY IN AN AGE-DEPENDENT MANNER IN FEMALE C57BL/6 MICE

**DOI:** 10.1093/geroni/igad104.3277

**Published:** 2023-12-21

**Authors:** Elise Bisset, Susan Howlett

**Affiliations:** Dalhousie University, Halifax, Nova Scotia, Canada; Dalhousie University, Halifax, Nova Scotia, Canada

## Abstract

We previously demonstrated that aerobic exercise attenuates the development of frailty in older female mice. Here we combine the common dietary supplement, sodium nitrate, with aerobic exercise to determine if this combination will work to attenuate frailty across lifespan. Adult (7-9 months) and aged (24-25 months) female mice were given free access to a running wheel and/or sodium nitrate (1mM, drinking water), or neither for 3 months. We measured blood pressure (tail cuff), running volume, frailty (frailty index), and body composition (DEXA). Blood pressure was unaffected by nitrates or exercise at both ages. While young mice ran more than older mice (3.5±0.4 vs 1.2±0.2 km/day), running was unaffected by nitrates. Older mice were frailer at baseline than younger mice (0.13±0.04 vs 0.03±0.004; p< 0.001). In older sedentary controls, frailty increased over time (0.15±0.02 to 0.19±0.03: p=0.03), but this was prevented by nitrates (0.13± 0.02 to 0.12 ± 0.01), exercise (0.12±0.03 to 0.18±0.03), and both (0.12±0.02 to 0.12±0.01). Older sedentary controls saw age-related weight loss (32.5±3.0g to 29.7±2.3g: p=0.02) which was prevented by exercise or nitrates. In older mice, mortality was highest in sedentary controls (46%) and lowest in exercised mice fed nitrates (0%). In contrast, while exercise attenuated frailty in young mice, nitrates did not. Young mice also had few changes in body composition; none died. While nitrates with or without exercise are beneficial for older mice, they had little effect in younger mice. These results suggest that nitrates alone or with exercise, may help prevent frailty in older females.

